# Breastfeeding and risk of food allergy and allergic rhinitis in offspring: a systematic review and meta-analysis of cohort studies

**DOI:** 10.1007/s00431-024-05580-w

**Published:** 2024-05-21

**Authors:** Yali Ding, Chengbi Zhu, Shuo Li, Naixu Liu, Qian Liu, Weifeng Li, Changjiang Zhao, Bin Yuan

**Affiliations:** 1 Department of Pediatrics, Affiliated Hospital of Nanjing University of Chinese Medicine, Nanjing Jiangsu, 210004 China; 2 Nanjing University of Chinese Medicine, Nanjing Jiangsu, 210023 China; 3 Nanjing Gaochun Traditional Chinese Medicine Hospital, Nanjing Jiangsu, 211300 China; 4 Department of Pediatrics, Jiangyin Affiliated Hospital of Nanjing University of Chinese Medicine, Jiangyin Jiangsu, 214400 China

**Keywords:** Children, Breastfeeding, Allergic rhinitis, Food allergy, Meta-analysis

## Abstract

The association between breastfeeding and the occurrence of allergic rhinitis (AR) and food allergy (FA) in offspring remains inconclusive. This review aims to comprehensively explore the potential relationships between various patterns and durations of breastfeeding and allergic diseases in offspring. We systematically searched PubMed, EMBASE, Cochrane, WOS databases, and Google Scholar for observational studies published up to March 30, 2023, that investigated the link between breastfeeding and allergies in offspring. The quality of the studies was assessed using the Newcastle-Ottawa Scale (NOS) and Joanna Briggs Institute (JBI). Pooled odds ratios (OR) and 95% confidence intervals (95% CI) were calculated employing an appropriate model based on the degree of heterogeneity. A total of 68 studies, encompassing 772,142 children, were ultimately included. The findings indicated that breastfeeding for more than 6 months was associated with a reduced risk of AR (OR = 0.88, 95% CI: 0.79 to 0.98) but posed a risk for FA (OR = 1.69, 95% CI: 1.27 to 2.25). Exclusive breastfeeding exhibited a protective effect against AR (OR = 0.94, 95% CI: 0.90 to 0.97), whereas non-breastfeeding was identified as a risk factor for AR (OR = 1.48; 95% CI: 1.03 to 2.12). No significant association was observed between breastfeeding patterns and FA.

* Conclusion*: Breastfeeding for more than 6 months proves to be an effective preventive measure against AR. However, large prospective high-quality studies are needed to investigate the potential risk of FA in children with prolonged breastfeeding.

**Table:** 

**What is Known:** *• The impact of breastfeeding on allergic rhinitis and food allergy in offspring is controversial.* *• Previous meta-analyses fail to prove the effect of breastfeeding on food allergy in offspring of all ages.*	
**What is New:** *• Breastfeeding for more than 6 months proves to be an effective preventive measure against AR. However, it potentially elevates the risk of FA in children. Non-breastfeeding is linked to an increased risk of AR in children, but there is no evidence of an association between breastfeeding patterns and FA in children.* *• The impact of breastfeeding on allergic rhinitis and food allergy in offspring may vary with the time and pattern of breastfeeding.*

**Supplementary Information:**

The online version contains supplementary material available at 10.1007/s00431-024-05580-w.

## Introduction

Allergic diseases have become increasingly prevalent among children, including conditions such as allergic rhinitis (AR), atopic dermatitis, bronchial asthma, and food allergy (FA) [1–4]. These conditions significantly impact the quality of life and pose an economic burden on affected children and their families [5], with potential life-threatening implications [6]. Kilanowski et al. [7] proposed that the surge in allergic disease prevalence in recent decades cannot be solely attributed to genetic factors. Akagawa et al. [8] also suggested a potential link to hygienic conditions, delivery methods, antibiotic usage, and Western diet improvements. How to prevent and manage allergic diseases in children has become a critical global public health concern.

In newborns, maintaining life after exposure to the extrauterine environment poses a major challenge to the immune system. The World Health Organization (WHO) recommends exclusive breastfeeding for infants under 6 months. As the primary source of natural nutrition for infants, breast milk provides not only essential nutrients such as protein and vitamins to support normal growth and development [9] but also white blood cells, antibodies, and immunoregulation factors to enhance the infant’s immune system [10]. Moreover, breastfed infants are generally healthier than formula-fed infants, with a significantly reduced risk of multiple serious diseases [11–14]. However, there is still a lack of in-depth and comprehensive research on the direct relationship between breastfeeding, childhood immunity, and allergic diseases.

In recent years, the association between breastfeeding and allergic diseases in children has become a focal point of research. Previous evidence-based medicine research has indicated that breastfeeding can reduce the risk of AR in children under 5 years old, but no significant association has been observed with their FA [15]. However, the limited number of included studies and substantial heterogeneity have diminished the reliability of this result. In their recent study, Hoang et al. found that long-term exclusive breastfeeding did not exhibit a protective effect against AR in children, with the overall evidence quality being relatively low [16]. Despite recent original studies exploring the link between breastfeeding and the risk of AR and FA, no conclusive findings have been drawn. Lyons et al. predicted factors contributing to FA in children and adults in Europe, revealing that breastfeeding for > 6 months served as a protective factor against FA [17]. In contrast, Yuenyongviwat et al., who studied risk factors for atopic dermatitis and FA in children, identified breastfeeding for more than 6 months as a major contributor to FA [18]. Saad et al. highlighted in their study on clinical phenotypes and related factors of milk allergy that exclusive breastfeeding was a protective factor against FA [19]. Matsumoto conducted a cohort study on nationwide births in Japan, concluding that exclusive breastfeeding was a risk factor for FA [20]. Tong et al., in a cross-sectional study on the prevalence and related risk factors of AR in children aged 6–12 years, found that breastfeeding for more than 6 months had a protective effect against AR [21]. However, Schmitz et al., who studied the prevalence and risk factors of atopic diseases in children and adolescents in Germany, identified breastfeeding for more than 6 months as a risk factor for AR [22]. The study by Rosas-Salazar et al. indicated that exclusive breastfeeding had a protective effect against rhinitis [23]. In contrast, Ahmed et al. demonstrated that exclusive breastfeeding promoted the occurrence of AR by evaluating eczema, asthma, AR, and allergic status in children in seventh graders from Iqaluit [24]. Given the limited and uncertain association between breastfeeding and allergic diseases in offspring, this systematic review and meta-analysis aim to synthesize existing evidence on the epidemiological characteristics of the relationship between breastfeeding and allergic diseases in offspring to quantify this association.

## Materials and methods

This report adheres to the guidelines outlined in the Preferred Reporting Items for Systematic Reviews and Meta-Analyses (PRISMA) [25] and Meta-analysis of Observational Studies in Epidemiology (MOOSE) [26]. Additionally, the meta-analysis was registered in PROSPERO (CRD42023416223).

### Search strategy

PubMed, Embase, Cochrane Library, Web of Science, and Google Scholar were comprehensively searched for English-language literature, from the inception of the databases to March 30, 2023. Our search employed a combination of subject terms and free-text terms, including key terms such as child, children, Breast Feeding, Breast Milk, Food Hypersensitivity, Allergic Rhinitis, among others. Table S1 provides detailed information on the search strategies. Additionally, we manually examined the reference lists of major studies and relevant review articles to identify any additional studies meeting our inclusion criteria.

### Literature screening

#### Inclusion criteria

(1) Participants: Offspring (with no age restriction); (2) Exposure factors: breastfeeding, measurement bases: questionnaires, follow-up, outpatient records, etc.; (3) Study types: observational study, including cross-sectional study, cohort study, case–control study; (4) Outcomes: AR, FA, with diagnostic criteria including Score for Allergic Rhinitis (SFAR), the International Study of Asthma and Allergies in Childhood (ISAAC), skin prick test (SPT), questionnaires and physician diagnosis, allergen-specific serum IgE levels, open food challenge test, and other criteria. (5) The study reported relative risk (RR), odds ratio (OR), and their 95% confidence interval (CIs) related to the outcome, or the original data were available for calculation.

#### Exclusion criteria

(1) Review, case report, research plan, or conference paper; (2) Clinical trial, animal, or in vitro study; (3) Duplicate publications and studies with no available full texts; (4) The literature did not provide information regarding the outcome indicators.

Literature screening was independently conducted by two reviewers (YL D and CB Z) based on the aforementioned criteria. Any disagreements during the screening process were addressed through discussion or, if necessary, by consulting a third reviewer (S L).

### Data extraction and quality assessment

Relevant data were independently extracted from each literature by two reviewers (YL D and CB Z). The extracted information included the first author, publication year, country, research type, basic information of subjects, exposure factors, setting of exposure level, outcome indicators, and adjusted confounding factors.

The quality of cohort studies and case-control studies was assessed by two independent reviewers using the Newcastle-Ottawa Scale (NOS) [27]. The evaluated aspects for cohort studies included the following eight domains: representativeness of the exposure cohort, selection of the non-exposure cohort, confirmation of exposure, certification that the results of concern did not exist at the beginning of the study, comparability of cohorts based on design or analysis, evaluation of outcomes, assessment of whether the duration of follow-up is sufficient to obtain outcomes, and adequacy of cohort follow-up. Similarly, the following eight domains were evaluated for case-control studies: adequacy of case definition, representativeness of cases, selection of controls, definition of controls, comparability of cases and controls based on design or analysis, determination of the extent of exposure, the shared method for determining cases and controls, and comparison of non-response rates in cases and controls. Each aspect, except for comparability, had a top score of 1, with comparability having a maximum score of 2. The studies were rated on a scale of 0–9. Therefore, a total score ≥ 6 points indicated high-quality research.

The risk of bias in cross-sectional studies was assessed using the Joanna Briggs Institute (JBI) critical appraisal checklist for analytical cross-sectional studies [28]. This tool consists of eight domains: sample inclusion, description of subjects and settings, valid and reliable measure of exposure, objective and standard measure of condition, identifying confounding factors, strategies to deal with confounding factors, valid and reliable measure of outcome, and appropriate statistical analysis. Each item can be answered by four response options: yes (1 score), no (0 score), unclear (0 score), or not applicable (0 score). Based on the items in the appraisal tool, the articles were categorized as high quality (80% score and above), moderate (60–80% score), and low quality (< 60% score).

### Data integration and statistical analysis

The primary outcome of interest was the relationship between breastfeeding and AR, while the secondary outcome was the association between breastfeeding and FA.

All meta-analyses utilized a random-effects model to combine effect sizes and were statistically analyzed using Stata 15.0, with a *P* value < 0.05 considered statistically significant. Heterogeneity across studies was assessed using Cochran’s *Q X*^2^ test, *I*^2^ statistic, and TAU (or tau-squared statistic), subgroup analysis and regression analysis were performed based on breastfeeding durations (< 4 months, 4–6 months, ≥ 6 months) and breastfeeding patterns (exclusive breastfeeding, partial breastfeeding, and non-breastfeeding) to determine heterogeneity and its sources among studies. Sensitivity analysis was used to assess the robustness of the meta-analysis by combining the results of the remaining studies after excluding each one individually [29, 30]. A funnel plot was generated to evaluate the potential publication bias in the included studies. Statistical tests (Egger’s or Begg’s test) were utilized to test the publication bias (at least eight studies were required). In case of significant publication bias, the trim-and-fill method was adopted to explore the impact of publication bias on the results.

## Results

### Literature screening results and flow chart

**Fig. 1:**
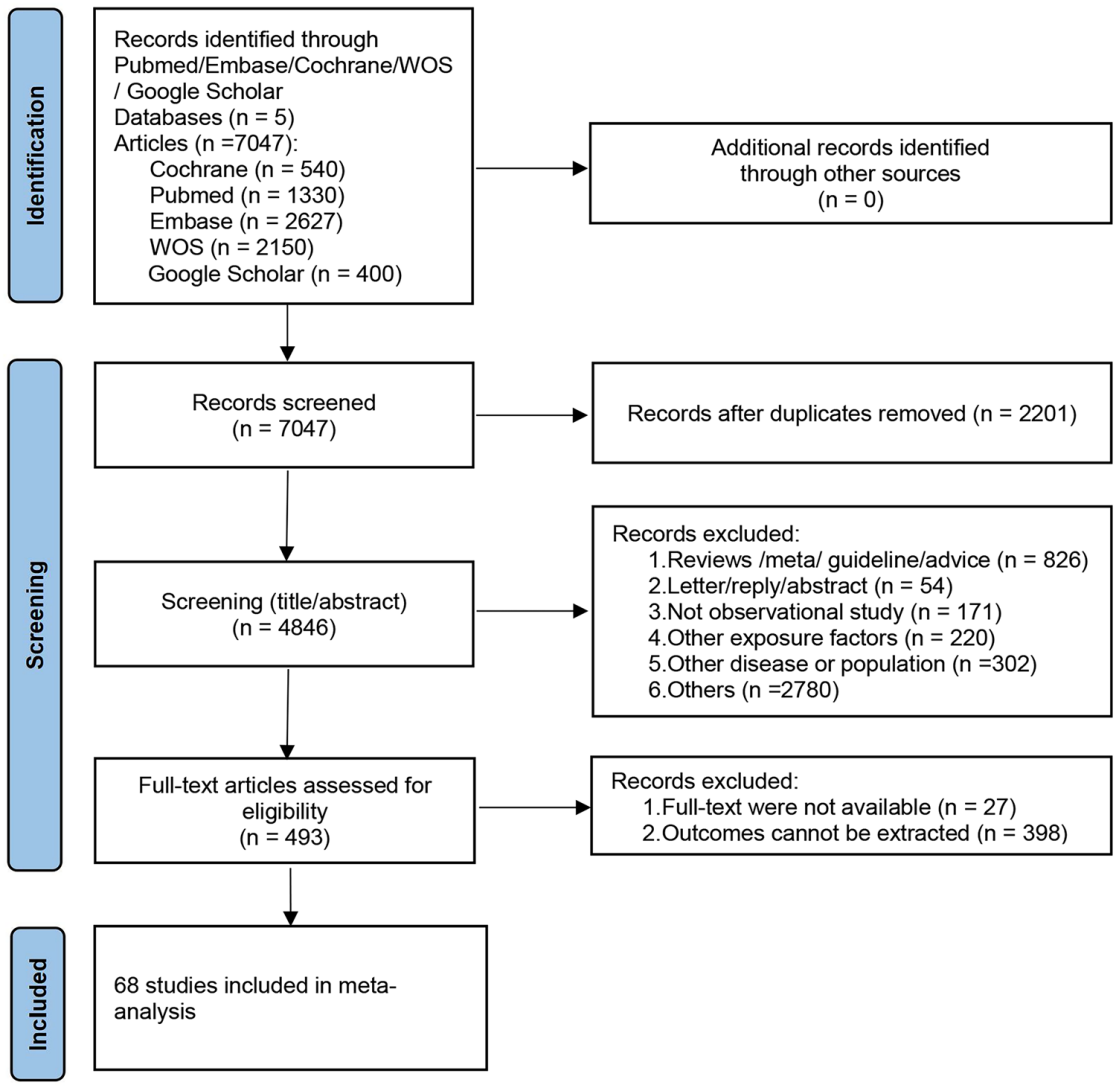
The PRISMA flowchart of the literature search and selection

A total of 7,047 papers were initially identified through the database search, and no additional research was discovered through the examination of references. Following the removal of duplicate literature, 4850 articles underwent title and abstract review. Among these, 4357 articles were excluded as they did not meet the inclusion criteria, leaving 493 articles for a thorough full-text review. Ultimately, this meta-analysis incorporated 68 studies [17–24, 31–90]. The literature screening process is illustrated in Fig. 1.

### Basic characteristics of included studies

The 68 included studies were conducted in 24 countries and regions, including China-Taiwan, China, Norway, Korea, Thailand, Turkey, Germany, Finland, Iran, Sweden, Canada, Japan, Netherlands, UK, America, Denmark, France, Egypt, Poland, Mexico, Kuwait, Spain, Australia, Qatar. In total, there were 35 articles on AR and 39 articles on FA. All studies were observational in nature, comprising cross-sectional studies (*n* = 30), cohort studies (*n* = 33), and case-control studies (*n* = 5). The participants across all publications were children, with a cumulative total of 772,142 children included in these studies. The sample size ranged from 65 to 46,616 in cohort studies, from 44 to 206,453 in cross-sectional studies, and from 126 to 422 in case-control studies. Most studies utilized the ISAAC questionnaires to assess allergic diseases, although other criteria such as skin prick tests, questionnaire surveys, and allergen-specific serum IgE levels were also employed to evaluate AR and FA. Additionally, potential confounding factors were adjusted in the majority of studies. The detailed characteristics of the included studies are illustrated in Table S2.

### Quality evaluation

The quality assessment using the NOS scale revealed that the scores of 38 included studies were all ≥ 6, as detailed in Table S3. Concerning subject selection, 32 studies scored 4, while 6 studies scored 3, indicating potential bias in results due to specific selection of the control population. Regarding comparability, all 33 studies scored 2, signifying that important confounding factors, including age, were controlled in each study. For outcome evaluation, 23 studies scored 3, and 15 studies scored 2, due to inaccurate outcome definition, nonuse of internationally standardized methods, insufficient follow-up time, and incomplete follow-up information. Among the 30 cross-sectional studies, 16 were rated as high quality, and 14 were considered medium quality, as outlined in Table S4.

### Meta-analysis results

#### Relationship between the duration of breastfeeding and allergic rhinitis in offspring

Twenty-two articles reported the relationship between the duration of breastfeeding and AR in offspring [21, 22, 31, 35–37, 42, 44, 51, 53, 56, 58–60, 63, 68, 70, 71, 77, 80, 83, 89]. The random effects model showed that there was no significant correlation between the duration of breastfeeding and AR in offspring (OR = 1.00, 95% CI:0.91 to 1.10, *P* = 0.995; *τ*^2^ = 0.0378, *I*^2^ = 91.0%, *P* < 0.001) (Fig. S1).

#### Relationship between the pattern of breastfeeding and allergic rhinitis in offspring

Sixteen articles investigated the relationship between breastfeeding patterns and AR in offspring [23, 24, 39, 50, 53, 56, 62, 64, 69, 72, 74, 75, 79–81, 90]. The random-effects model indicated no significant correlation between breastfeeding patterns and AR in offspring (OR = 1.06, 95% CI: 0.96 to 1.6, *P* = 0.238; *τ*^2^ = 0.0233, *I*^2^ = 89.5%, *P* < 0.001) (Fig. S2).

#### Relationship between the duration of breastfeeding and food allergy in offspring

Twenty-four articles reported the relationship between the duration of breastfeeding and FA in offspring [17, 18, 20, 33, 35, 36, 38, 40, 41, 45, 48, 49, 54, 55, 57, 61, 65–67, 73, 76, 82, 86, 87]. The random effects model showed that there was no significant correlation between the duration of breastfeeding and FA in offspring (OR = 1.37, 95% CI: 1.11 to 1.68, *P* = 0.004; *τ*^2^ = 0.1614, *I*^2^ = 78.9%, *P* < 0.001) (Fig. S3).

#### Relationship between the pattern of breastfeeding and food allergy in offspring

Twenty articles reported the relationship between the pattern of breastfeeding and FA in offspring [19, 20, 23, 24, 32, 34, 43, 45–47, 49, 50, 52, 55, 73, 78, 79, 84, 85, 88]. There was no significant correlation between the pattern of breastfeeding and FA in offspring (OR = 1.00, 95% CI: 0.85 to 1.19, *P* = 0.957; *τ*^2^ = 0.1071, *I*^2^ = 79.2%, *P* < 0.001) (Fig. S4).

### Subgroup analysis and regression analysis

**Fig. 2:**
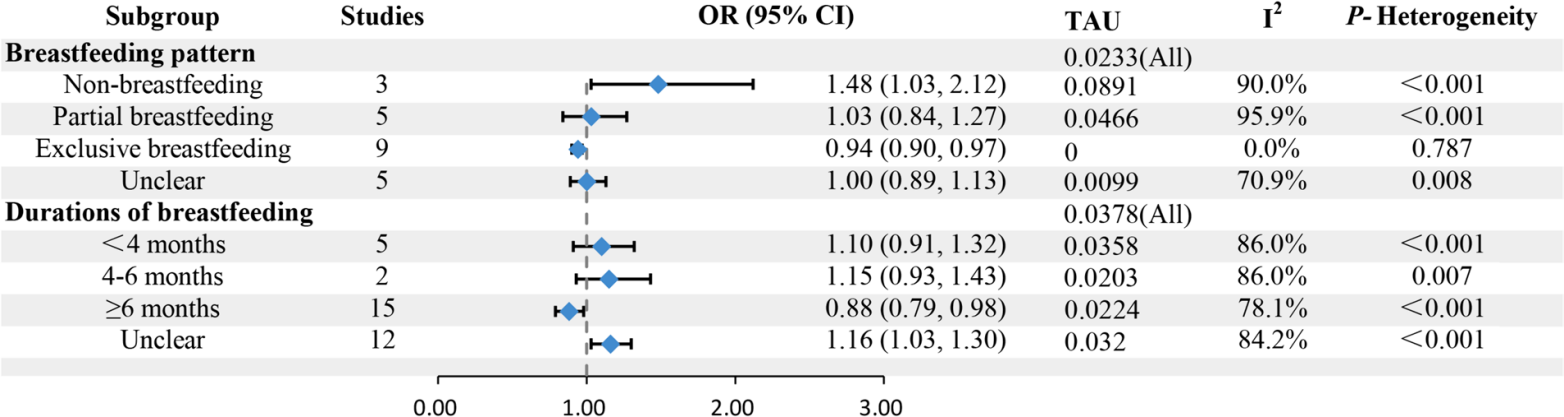
Subgroup analysis of the association between breastfeeding and allergic rhinitis based on breastfeeding pattern and duration

**Fig. 3:**
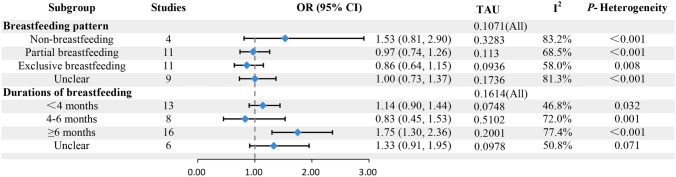
Subgroup analysis of the association between breastfeeding and food allergy based on breastfeeding pattern and duration

We performed a subgroup analysis for AR and FA based on the durations of breastfeeding (< 4 months, 4–6 months, ≥ 6 months) and breastfeeding patterns (exclusive breastfeeding, partial breastfeeding, and non-breastfeeding) to investigate the sources of heterogeneity (Figs. 2 and 3).

### Association between different durations of breastfeeding and allergy in offspring

After conducting a subgroup analysis to assess the impact of different breastfeeding durations on AR, the results indicated a significant reduction in the risk of AR in offspring with breastfeeding for more than 6 months (OR = 0.88, 95% CI: 0.79 to 0.98; *P* = 0.015). However, there was no significant correlation observed between breastfeeding durations of < 4 months or 4–6 months and AR in offspring (OR = 1.10, 95% CI: 0.91 to 1.32; OR = 1.15, 95% CI: 0.93 to 1.43; respectively). Regression analysis demonstrated no significant difference in the impact of different breastfeeding durations on the extent of the correlation (*P* = 0.790).

Following the subgroup analysis of the influence of different breastfeeding durations on FA, the results revealed a significant increase in the risk of FA in offspring with breastfeeding for more than 6 months (OR = 1.75, 95% CI: 1.30 to 2.36). Conversely, there was no significant correlation found between breastfeeding durations of < 4 months or 4–6 months and FA in offspring (OR = 1.14, 95% CI: 0.90 to 1.44; OR = 0.83, 95% CI: 0.45 to 1.53; respectively). Additionally, regression analysis indicated no significant difference in the protective effect of different breastfeeding durations against FA in offspring (*P* = 0.126).

### Relationship between different patterns of breastfeeding and allergy in offspring

After conducting a subgroup analysis to assess the influence of different patterns of breastfeeding on AR, the results indicated that non-breastfeeding was a risk factor for AR in offspring (OR = 1.48; 95% CI: 1.03 to 2.12), while exclusive breastfeeding had a protective effect against AR in offspring (OR = 0.94, 95% CI: 0.90 to 0.97). No significant correlation was observed between partial breastfeeding and AR in offspring (OR = 1.03; 95% CI: 0.84 to 1.27). Additionally, regression analysis revealed a significant difference in the protective effect of different patterns of breastfeeding against AR in offspring (*P* = 0.026), suggesting that the variation in breastfeeding patterns may contribute to heterogeneity.

Following the subgroup analysis of the influence of different patterns of breastfeeding on FA, the results indicated no significant correlation between non-breastfeeding, partial breastfeeding, or exclusive breastfeeding, and FA in offspring (OR = 1.53, 95% CI: 0.81 to 2.90; OR = 0.97, 95% CI: 0.74 to 1.26; OR = 0.86, 95% CI: 0.64 to 1.15; respectively). In regression analysis, there was no significant difference in the protective effect of different patterns of breastfeeding against FA in offspring (*P* = 0.324).

### Sensitivity analysis

Sensitivity analyses were conducted separately for the influences of the duration and pattern of breastfeeding on AR and FA. The impact of each study on the summarized results was evaluated using a one-by-one exclusion method. The analysis results demonstrated that none of the pooled results were significantly affected by any individual study. This suggested that the results of this meta-analysis were relatively reliable (Fig. S5).

### Publication bias

To ensure the validity of the meta-analysis results, we employed a funnel plot and conducted Egger’s and Begg’s tests to assess the publication bias for primary outcome indicators. The results indicated that there was no significant publication bias in the four outcome indicators (*P* > 0.05).

## Discussion

Breast milk is explicitly acknowledged by the World Health Organization (WHO) as the preferred nourishment for newborns, even during public health crises [91]. It serves as a primary source of nutrition and energy for the early growth and development of infants. Global statistics indicate that enhancing the worldwide breastfeeding rate could potentially save the lives of over 820,000 children under 5 years old annually. However, this optimal feeding practice has not received adequate attention in high-income countries [9]. This may be attributed to their rapid socio-economic development, accelerated pace of life, and escalating pressures from family and work. For children, improved economic conditions provide the foundation for a better life, but the initial provision of adequate and scientifically sound nutrition is the cornerstone of lifelong health. This underscores the significance of prioritizing breastfeeding.

Our study findings underscore that breastfeeding for more than 6 months is effective in preventing AR, whereas non-breastfeeding increases the risk of AR in children. We observed a significant association between the duration of breastfeeding and FA in offspring. Prolonged breastfeeding (≥ 6 months), however, was linked to an increased risk of FA in children. Importantly, no evidence was found to suggest an association between the pattern of breastfeeding and FA in children.

Dr. Gabryszewski, in their study, reported a positive impact of breastfeeding in reducing the incidence of AR in children [92]. Our subgroup analysis results align with this, indicating that breastfeeding for more than 6 months protects against AR in offspring. This finding is consistent with the World Health Organization’s (WHO) recommendation of breastfeeding for at least 6 months [9]. Li et al. [93] discovered that in the comparison of children with house dust mite-induced allergic rhinitis (HDM-AR) and healthy controls, among children carrying the protective TT genotype of IL18R1_rs2287037, those exclusively breastfed for the first 4 months had a significantly reduced risk of rhinitis (OR = 0.33). This may offer insights into bioinformatics research on AR. On the contrary, non-breastfeeding emerged as a risk factor for AR in offspring. Studies have indicated that early respiratory tract infections under the age of 2 are closely linked to AR in children [94]. Various bioactive molecules in breast milk are effective in preventing early infections in children [10]. While this provides a plausible explanation for our results, further evidence is required to substantiate these findings due to the limited data in this subgroup.

FA in children is considered the “second epidemic wave” following asthma and AR [95]. Its prevalence is gradually increasing, with the severity of allergic reactions also on the rise [96]. Our results indicate that breastfeeding for over 6 months is a risk factor for FA in offspring. This finding can be explained by dietary diversity, which is defined as the variety of foods or food combinations consumed within a specific timeframe [97]. Changes in diet significantly impact intestinal microorganisms [98]. It has been shown that a diversified diet in infancy can increase butyrate, helping reduce the risk of allergies in children [99]. Butyrate promotes the extratymatic generation of Treg cells, maintains the intestinal barrier, and supports the development and function of the immune system [100–102]. Additionally, early introduction of allergic foods is considered as a primary method for preventing food allergies. Current guidelines [103] recommend regular and long-term complementary feeding for high-risk allergy children after systematic evaluation by physicians based on specific IgE levels and skin prick tests. This induces immune tolerance in the body. Prescott [104] highlighted the age of 4 to 6 months as a key period for establishing immune tolerance in early life. Reasonable introduction of complementary foods at this age is beneficial for preventing allergic diseases in infants. A 2016 randomized trial assessing the preventive effect of early introduction of six allergic foods (cow’s milk, hen’s egg, peanut, sesame, codfish, and heat) on FA demonstrated a 67% decrease in the relative risk of FA in children intaking allergic foods early [105].

This provides new ideas for us to explore why breastfeeding for more than 6 months increases the risk of FA. However, we believe it may be premature to draw conclusions based on the existing evidence. In addition to prolonged breastfeeding time, FA may also be caused by late introduction of solid food during this period. Hence, late introduction of solid food may play a pivotal role in the development of FA [106]. Recommendations released in 2022 also strongly recommend the introduction of potentially allergenic foods in infants at 6 months of age, without postponing or bringing exposure forward to reduce the risk of FA [107]. Therefore, if solid foods are not introduced when breastfeeding is prolonged, infants may lose a crucial window of opportunity to develop tolerance to potential allergens. Allergic diseases in children are undeniably complex, diverse, and multifaceted. Our study faced inevitable high heterogeneity in our statistics, particularly among children who received breastfeeding for 6–12 months. Given the differences in the duration of feeding and the limited number of studies in subgroups, further subdivision is difficult. Therefore, this seemingly contradictory statistical result still requires more supportive data. Additionally, we did not explore national differences in published studies, and individual factors such as the child’s gender, age, and geographical location might contribute to high heterogeneity in statistical results.

## Strengths and limitations

Our research can be considered as an update to previous evidence. First, we included more recent literature on breastfeeding and the risk of AR and FA, and most included studies are considered of high quality. Still, the reliability of data was reduced in a few studies due to inaccurate definitions of outcomes and a lack of follow-ups. Second, since these studies are observational, all participants are unlikely to be interfered with by investigators. During the breastfeeding period described, if allergic symptoms or early signs were observed in children, parents might briefly stop breastfeeding and choose corresponding drug interventions, which could cause discontinuity of exposure factors and result in biased results. Additionally, due to the high proportion of retrospective studies included and the variations in the number and types of adjustment factors, it may be inevitable to avoid the influence of recall bias and other negative factors. A more rigorous assessment is required to eliminate these influences, such as the establishment of uniform diagnostic criteria for allergic diseases, and acquisition of more detailed records and reports to balance the feeding dose of breast milk. Finally, we did not explore the relationship between breastfeeding for more than 12 months and allergy in offspring due to insufficient data. Despite these shortcomings, the likelihood of publication bias in our study is low, suggesting that our summarized evidence is reliable overall.

## Conclusion

This meta-analysis reveals that non-breastfeeding increases the risk of AR in children, whereas breastfeeding for more than 6 months effectively prevents AR, aligning with the regimen recommended by WHO. Regarding FA, there is no evidence to suggest an association between the pattern of breastfeeding and FA in children. However, there is a significant correlation between the duration of breastfeeding and FA. Breastfeeding for more than 6 months increases the risk of FA in children, possibly due to the lack of a diversified diet hindering the development of early immunity. Due to the intricate nature of breastfeeding and the limitations of the types of included studies, further improvements and in-depth research are needed in the future.

### Supplementary Information

Below is the link to the electronic supplementary material.

Supplementary file1 (DOCX 1597 KB)

## Data Availability

Data sharing not applicable to this article as no datasets were generated or analysed during the current study.

## Abbreviations

AR: Allergic rhinitis
FA: Food allergy
HDM-AR: House dust mite-induced allergic rhinitis
ISAAC: International Study of Asthma and Allergies in Childhood
JBI: Joanna Briggs Institute
MOOSE: Meta-analysis of Observational Studies in Epidemiology
NOS: Newcastle-Ottawa Scale
OR: Odds ratios
PRISMA: Preferred Reporting Items for Systematic Reviews and Meta-Analyses
RR: Relative risk
SFAR: Score for Allergic Rhinitis
SPT: Skin prick test
WHO: World Health Organization
95% CI: 95% Confidence intervals
