# Supplementary material for: Breastfeeding and risk of food allergy and allergic rhinitis in offspring: a systematic review and meta-analysis of cohort studies
Source: Eur J Pediatr. 2024 May 21;183(8):3433–43. doi: 10.1007/s00431-024-05580-w (PMC11263247; doi:10.1007/s00431-024-05580-w)
Supplement: Supplementary file 1 — Supplementary file1 (DOCX 1597 KB) [file 431_2024_5580_MOESM1_ESM.docx]

**Table S1:** Literature search strategy.

**1.Pubmed**

| Search number | Query |
| --- | --- |
| #1 | "Child"[Mesh] |
| #2 | ((((((((Child[Title/Abstract]) OR (children[Title/Abstract])) OR (childhood[Title/Abstract])) OR (infant[Title/Abstract])) OR (newborn[Title/Abstract])) OR (preschool child[Title/Abstract])) OR (kid[Title/Abstract])) OR (kids[Title/Abstract])) OR (offspring[Title/Abstract]) |
| #3 | ("Child"[Mesh]) OR (((((((((Child[Title/Abstract]) OR (children[Title/Abstract])) OR (childhood[Title/Abstract])) OR (infant[Title/Abstract])) OR (newborn[Title/Abstract])) OR (preschool child[Title/Abstract])) OR (kid[Title/Abstract])) OR (kids[Title/Abstract])) OR (offspring[Title/Abstract])) |
| #4 | "Breast Feeding"[Mesh] |
| #5 | (((((((((((Breast Feeding[Title/Abstract]) OR (breastfeeding[Title/Abstract])) OR (Breast Fed[Title/Abstract])) OR (Breastfed[Title/Abstract])) OR (breast milk[Title/Abstract])) OR (human milk[Title/Abstract])) OR (infant formula[Title/Abstract])) OR (infant nutrition[Title/Abstract])) OR (Wet Nursing[Title/Abstract])) OR (Milk Sharing[Title/Abstract])) OR (breast milk expression[Title/Abstract])) OR (Exclusive Breastfeeding[Title/Abstract]) |
| #6 | ("Breast Feeding"[Mesh]) OR ((((((((((((Breast Feeding[Title/Abstract]) OR (breastfeeding[Title/Abstract])) OR (Breast Fed[Title/Abstract])) OR (Breastfed[Title/Abstract])) OR (breast milk[Title/Abstract])) OR (human milk[Title/Abstract])) OR (infant formula[Title/Abstract])) OR (infant nutrition[Title/Abstract])) OR (Wet Nursing[Title/Abstract])) OR (Milk Sharing[Title/Abstract])) OR (breast milk expression[Title/Abstract])) OR (Exclusive Breastfeeding[Title/Abstract])) |
| #7 | (("Food Hypersensitivity"[Mesh])) OR "Rhinitis, Allergic"[Mesh] |
| #8 | (((((((((((Food Hypersensitivity[Title/Abstract]) OR (Rhinitis, Allergic[Title/Abstract])) OR (Food Hypersensitivities[Title/Abstract])) OR (Food Allergy[Title/Abstract])) OR (Food Allergies[Title/Abstract])) OR (Allergy Food[Title/Abstract])) OR (Allergic Rhinitides[Title/Abstract])) OR (Allergic Rhinitis[Title/Abstract])) OR (allergic rhinopathy[Title/Abstract])) OR (atopic rhinitis[Title/Abstract])) OR (rhinitis allergica[Title/Abstract])) OR (rhinitis atopica[Title/Abstract]) |
| #9 | ((("Food Hypersensitivity"[Mesh])) OR "Rhinitis, Allergic"[Mesh]) OR ((((((((((((Food Hypersensitivity[Title/Abstract]) OR (Rhinitis, Allergic[Title/Abstract])) OR (Food Hypersensitivities[Title/Abstract])) OR (Food Allergy[Title/Abstract])) OR (Food Allergies[Title/Abstract])) OR (Allergy Food[Title/Abstract])) OR (Allergic Rhinitides[Title/Abstract])) OR (Allergic Rhinitis[Title/Abstract])) OR (allergic rhinopathy[Title/Abstract])) OR (atopic rhinitis[Title/Abstract])) OR (rhinitis allergica[Title/Abstract])) OR (rhinitis atopica[Title/Abstract])) |
| #10 | ((("Child"[Mesh]) OR (((((((((Child[Title/Abstract]) OR (children[Title/Abstract])) OR (childhood[Title/Abstract])) OR (infant[Title/Abstract])) OR (newborn[Title/Abstract])) OR (preschool child[Title/Abstract])) OR (kid[Title/Abstract])) OR (kids[Title/Abstract])) OR (offspring[Title/Abstract]))) AND (("Breast Feeding"[Mesh]) OR ((((((((((((Breast Feeding[Title/Abstract]) OR (breastfeeding[Title/Abstract])) OR (Breast Fed[Title/Abstract])) OR (Breastfed[Title/Abstract])) OR (breast milk[Title/Abstract])) OR (human milk[Title/Abstract])) OR (infant formula[Title/Abstract])) OR (infant nutrition[Title/Abstract])) OR (Wet Nursing[Title/Abstract])) OR (Milk Sharing[Title/Abstract])) OR (breast milk expression[Title/Abstract])) OR (Exclusive Breastfeeding[Title/Abstract])))) AND (((("Food Hypersensitivity"[Mesh])) OR "Rhinitis, Allergic"[Mesh]) OR ((((((((((((Food Hypersensitivity[Title/Abstract]) OR (Rhinitis, Allergic[Title/Abstract])) OR (Food Hypersensitivities[Title/Abstract])) OR (Food Allergy[Title/Abstract])) OR (Food Allergies[Title/Abstract])) OR (Allergy Food[Title/Abstract])) OR (Allergic Rhinitides[Title/Abstract])) OR (Allergic Rhinitis[Title/Abstract])) OR (allergic rhinopathy[Title/Abstract])) OR (atopic rhinitis[Title/Abstract])) OR (rhinitis allergica[Title/Abstract])) OR (rhinitis atopica[Title/Abstract]))) |

**2.Cochrane**

| Search number | Query |
| --- | --- |
| #1 | MeSH descriptor: [Child] explode all trees |
| #2 | (Child):ti,ab,kw OR (children):ti,ab,kw OR (childhood):ti,ab,kw OR (infant):ti,ab,kw OR (newborn):ti,ab,kw |
| #3 | (preschool child):ti,ab,kw OR (kid):ti,ab,kw OR (kids):ti,ab,kw OR (offspring):ti,ab,kw |
| #4 | #1 or #2 or #3 |
| #5 | MeSH descriptor: [Breast Feeding] explode all trees |
| #6 | (Breast Feeding):ti,ab,kw OR (breastfeeding):ti,ab,kw OR (Breast Fed):ti,ab,kw OR (Breastfed):ti,ab,kw OR (breast milk):ti,ab,kw |
| #7 | (human milk):ti,ab,kw OR (infant formula):ti,ab,kw OR (infant nutrition):ti,ab,kw OR (Wet Nursing):ti,ab,kw OR (Milk Sharing):ti,ab,kw |
| #8 | (breast milk expression):ti,ab,kw OR (Exclusive Breastfeeding):ti,ab,kw |
| #9 | #5 or #6 or #7 or #8 |
| #10 | MeSH descriptor: [Food Hypersensitivity] explode all trees |
| #11 | MeSH descriptor: [Rhinitis, Allergic] explode all trees |
| #12 | #10 or #11 |
| #13 | (Food Hypersensitivity):ti,ab,kw OR (Rhinitis, Allergic):ti,ab,kw OR (Food Hypersensitivities):ti,ab,kw OR (Food Allergy):ti,ab,kw OR (Food Allergies):ti,ab,kw |
| #14 | (Allergy Food):ti,ab,kw OR (Allergic Rhinitides):ti,ab,kw OR (Allergic Rhinitis):ti,ab,kw OR (allergic rhinopathy):ti,ab,kw OR (atopic rhinitis):ti,ab,kw |
| #15 | (rhinitis allergica):ti,ab,kw OR (rhinitis atopica):ti,ab,kw |
| #16 | #12 or #13 or #14 or #15 |
| #17 | #4 AND #9 AND #16 |

**3.Embase**

| Search number | Query |
| --- | --- |
| #1 | 'child'/exp |
| #2 | child:ab,ti OR children:ab,ti OR childhood:ab,ti OR infant:ab,ti OR newborn:ab,ti OR 'preschool child':ab,ti OR kid:ab,ti OR kids:ab,ti OR offspring:ab,ti |
| #3 | #1 OR #2 |
| #4 | 'breast feeding'/exp |
| #5 | 'breast feeding':ab,ti OR breastfeeding:ab,ti OR 'breast fed':ab,ti OR breastfed:ab,ti OR 'breast milk':ab,ti OR 'human milk':ab,ti OR 'infant formula':ab,ti OR 'infant nutrition':ab,ti OR 'wet nursing':ab,ti OR 'milk sharing':ab,ti OR 'breast milk expression':ab,ti OR 'exclusive breastfeeding':ab,ti |
| #6 | #4 OR #5 |
| #7 | 'food allergy'/exp |
| #8 | 'allergic rhinitis'/exp |
| #9 | #7 OR #8 |
| #10 | 'food hypersensitivity':ab,ti OR 'rhinitis, allergic':ab,ti OR 'food hypersensitivities':ab,ti OR 'food allergy':ab,ti OR 'food allergies':ab,ti OR 'allergy food':ab,ti OR 'allergic rhinitides':ab,ti OR 'allergic rhinitis':ab,ti OR 'allergic rhinopathy':ab,ti OR 'atopic rhinitis':ab,ti OR 'rhinitis allergica':ab,ti OR 'rhinitis atopica':ab,ti |
| #11 | #9 OR #10 |
| #12 | #3 AND #6 AND #11 |

**4.Web of science**

| Search number | Query |
| --- | --- |
| #1 | TS=(Child) OR TS=(children) OR TS=(childhood) OR TS=(infant) OR TS=(newborn) OR TS=(preschool child) OR TS=(kid) OR TS=(kids) OR TS=(offspring) |
| #2 | TS=(Breast Feeding) OR TS=(breastfeeding) OR TS=(Breast Fed) OR TS=(Breastfed) OR TS=(breast milk) OR TS=(human milk) OR TS=(infant formula) OR TS=(infant nutrition) OR TS=(Wet Nursing) OR TS=(Milk Sharing) OR TS=(breast milk expression) OR TS=(Exclusive Breastfeeding) |
| #3 | TS=(Food Hypersensitivity) OR TS=(Rhinitis, Allergic) OR TS=(Food Hypersensitivities) OR TS=(Food Allergy) OR TS=(Food Allergies) OR TS=(Allergy Food) OR TS=(Allergic Rhinitides) OR TS=(Allergic Rhinitis) OR TS=(allergic rhinopathy) OR TS=(atopic rhinitis) OR TS=(rhinitis allergica) OR TS=(rhinitis atopica) |
| #4 | #3 AND #2 AND #1 |

**5.Google Scholar**

| Search number | Query |
| --- | --- |
| #1 | (Child OR children OR childhood OR infant OR newborn OR "preschool child" OR kid OR kids OR offspring) AND ("Breast Feeding" OR breastfeeding OR "breast milk" OR "human milk" OR "infant formula" OR "infant nutrition" OR "Wet Nursing" OR "Milk Sharing" OR "breast milk expression" OR "Exclusive Breastfeeding") AND ("Food Hypersensitivity" OR "Food Hypersensitivities" OR "Allergic Rhintis" OR "Food Allergy" OR "Food Allergies" OR "Allergy Food" OR "Allergic Rhinitis" OR "allergic rhinopathy" OR "atopic rhinitis" OR "rhinitis allergica" OR "rhinitis atopica") |

**Table S2:** Characteristics of the included studies.

| No. | First Author | Year | Country | Study design | Sample size (Child) | Male/Female (Child) | Age, year (Child) | Feeding pattern |
| --- | --- | --- | --- | --- | --- | --- | --- | --- |
| 1 | Tong et al. | 2022 | China | Cross-sectional study | 10757 | 5809/4948 | 9.12±1.76 years | BF |
| 2 | Ekelund et al. | 2021 | Norway | Cohort study | 6796 | 3379/3415 | 1.7-7.5 years | BF |
| 3 | Tong et al. | 2020 | China | Cross-sectional study | 5550 | 2993/2557 | 8.93±1.741 years | BF |
| 4 | Yu et al. | 2019 | China | Cross-sectional study | 183449 | 98565/84884 | 9.94±2.99 years | BF, Exclusive BF, Never BF |
| 5 | Han et al. | 2019 | Korea | Cross-sectional study | 1374 | 941/433 | 4-12 years | BF |
| 6 | Chinratanapisit et al. | 2019 | Thailand | Cross-sectional study | 6291 | 3278/3013 | 6-7 years | BF |
| 7 | Lee et al. | 2017 | Taiwan,China | Cohort study | 1848 | 926/817 (6y) | 3,6 years | BF |
| 8 | Huang et al. | 2017 | China | Cross-sectional study | 13335 | 6536/6753 | 4-6 years | BF, Exclusive BF, Partial BF |
| 9 | Wang et al. | 2016 | China | Cross-sectional study | 13335 | NM | 4-6 years | BF, Exclusive BF, Never BF |
| 10 | Chiu et al. | 2016 | Taiwan,China | Cohort study | 186 | 83/103 | 1-4 years | BF |
| 11 | Wang et al. | 2015 | China | Cross-sectional study | 5388 | 2839/2549 | 3-6 years | BF |
| 12 | Li et al. | 2015 | China | Cross-sectional study | 20803 | 10311/10492 | 5-13 years | Exclusive BF |
| 13 | Tamay et al. | 2014 | Turkey | Cross-sectional study | 9875 | 4972/4835 | 6-7 years | BF |
| 14 | Song et al. | 2014 | China | Cross-sectional study | 10338 | 5095/5243 | 6-18 years | BF |
| 15 | Schmitz et al. | 2012 | Germany | Cross-sectional study | 17450 | 8880/8570 | 0-17 years | Exclusive BF |
| 16 | Kellberger et al. | 2012 | Germany | Cohort study | 3785 | 1802/1983 | 9-11 years | Exclusive BF, Never BF |
| 17 | Chen et al. | 2012 | Taiwan,China | Cross-sectional study | 2749 | 1367/1324 | 6-14 years | BF |
| 18 | Siriaksorn et al. | 2011 | Thailand | Cross-sectional study | 2301 | NM | 4.44±0.84 years | Exclusive BF |
| 19 | Sandini et al. | 2011 | Finland | Cohort study | 934 | 464/470 | 2,5 years | Exclusive BF, BF |
| 20 | Sahebi et al. | 2011 | Iran | Cross-sectional study | 1508 | 793/715 | 12.56 years | BF |
| 21 | Bjorksten et al. | 2011 | Sweden | Cross-sectional study | 206453 | NM | 6-7 years | BF |
| 22 | Kramer et al. | 2009 | Canada | Cohort study | 13889 | 7181/6708 | 6.5 years | Exclusive BF |
| 23 | Miyake et al. | 2007 | Japan | Cross-sectional study | 20477 | 11916/12161 | 6-15 years | Exclusive BF, Partial BF |
| 24 | Kurt et al. | 2007 | Turkey | Cross-sectional study | 25843 | 12931/12912 | 9.5±1.7 years | BF |
| 25 | Obihara et al. | 2005 | Netherlands | Cross-sectional study | 861 | 438/423 | 10.4±2.5 years | BF |
| 26 | Butland et al. | 1997 | UK | Cohort study | 20582 | 10304/10302 | 16 years | BF |
| 27 | Strachan et al. | 1996 | UK | Cohort study | 11765 | 5780/5985 | 11-16 years | BF |
| 28 | Rosas-Salazar et al. | 2022 | America | Cohort study | 1949 | 1020/929 | 55 (16-78)days-4years | Exclusive BF, Partial BF, Never BF |
| 29 | Huang et al. | 2021 | Taiwan,China | Cross-sectional study | 3192 | 1725/1467 | NM | BF |
| 30 | Hu et al. | 2021 | China | Cross-sectional study | 10464 | 5464/5000 | 9.2±2.2 years | BF |
| 31 | Ahmed et al. | 2019 | Canada | Cross-sectional study | 58 | 28/30 | 13-14 years | Exclusive BF |
| 32 | Ahmed et al. | 2018 | Canada | Cross-sectional study | 44 | 26/18 | 6-7 years | Exclusive BF |
| 33 | Jelding-Dannemand et al. | 2015 | Denmark | Cohort study | 389 | 189/200 | 0.5-6 years | Exclusive BF |
| 34 | Kusunoki et al. | 2010 | Japan | Cross-sectional study | 13110 | 6655/6455 | 7-15 years | Exclusive BF |
| 35 | Ehlayel et al. | 2008 | State of Qatar | Cross-sectional study | 1278 | 632/646 | 0-5 years | Exclusive BF |
| 36 | Sung et al. | 2022 | China | Cohort study | 183 | NM | 4 years | Partial BF |
| 37 | Yuenyongviwat et al. | 2021 | Thailand | Cross-sectional study | 119 | 75/44 | 8 (2, 69)months | BF |
| 38 | Pelak et al. | 2021 | USA | Cohort study | 285 | 150/135 | 12 months | Partial BF |
| 39 | Lang et al. | 2021 | USA | Cohort study | 1427 | NM | 0-21 years | BF |
| 40 | Amazouz et al. | 2021 | France | Cohort study | 3840 | 1971/1870 | 0-8 years | Exclusive BF |
| 41 | Saad et al. | 2020 | Egypt | Cross-sectional study | 317 | 175/142 | 4.2 ± 3.2 months | Partial BF |
| 42 | Matsumoto et al. | 2020 | Janpan | Cohort study | 46616 | 24220/22396 | NM | Partial BF |
| 43 | Lyons et al. | 2020 | The Netherlands | Cross-sectional study | 2196 | 1157/1169 | 7-10 years | BF |
| 44 | Sicherer et al. | 2019 | USA | Cohort study | 511 | 345/166 | 9.9 (3.1-15.0) months | BF |
| 45 | Kim et al. | 2019 | Korea | Cohort study | 1628 | NM | 33 (31-35) months | BF |
| 46 | Davisse-Paturet et al. | 2019 | France | Cohort study | 17574 | 5965/5755 (No loss) | NM | Partial BF |
| 47 | Wang et al. | 2018 | China | Cross-sectional study | 1008 | NM | 0-18 years | Partial BF |
| 48 | van Ginkel et al. | 2018 | The Netherlands | Retrospective cohort study | 724 | 399/325 | 5-214 months | BF |
| 49 | Sardecka et al. | 2018 | Poland | Case control study | Case group: 138 Control group: 101 | Case group: 74/64 Control group: 52/49 | Case group: 7.4±7.2 years Control group: 8.5±6.2 years | Partial BF |
| 50 | Bedolla-Barajas et al. | 2018 | Mexico | Case control study | Case group: 97 Control group: 97 | Case group: 63/34 Control group: 63/34 | Case group: 3.6 ± 1.2 years Control group: 3.7 ± 1.3 years | Partial BF |
| 51 | Alkazemi et al. | 2018 | Kuwait | Case control study | FA: 100 Control group: 100 | FA:67/33 Control:55/45 | NM | Exclusive BF |
| 52 | Jonsson et al. | 2017 | Sweden | birth cohort study | 65 | 33/32 | NM | Exclusive BF |
| 53 | Gil et al. | 2017 | Spain | case control study | Case group: 211 Control group: 211 | Case group: 128/83 Control group: 128/83 | Case group: 14.41 ± 5.42 years Control group: 14.59 ± 4.09 years | Partial BF |
| 54 | Elbert et al. | 2017 | The Netherlands | prospective cohort study | 5828 | 2909/2919 | 9.7 years | Partial BF |
| 55 | Goldsmith et al. | 2016 | Australia | Cohort study | 4537 | 2314/2220 | 1 years | Exclusive BF |
| 56 | Kim et al. | 2015 | South Korea | Case control study | 126 | 75/51 | 1.6±1.6 years | Exclusive BF |
| 57 | Luccioli et al. | 2014 | USA | Cohort study | 1363 | 688/675 | 0-12 months | Exclusive BF |
| 58 | Nwaru et al. | 2013 | Finland | birth cohort study | 3675 | 1930/1745 | 0-5 years | Exclusive BF |
| 59 | Hong et al. | 2011 | USA | Cohort study | 970 | 480/484 | 2.5 ± 2.2 years | Exclusive BF |
| 60 | Sanchez-Valverde et al. | 2009 | Spain | prospective cohort study | 225 | 122/103 | NM | BF |
| 61 | Kljakovic et al. | 2009 | Australian | Cross-sectional study | 3739 | 1880/1859 | 5 years | BF |
| 62 | Pesonen et al. | 2006 | Finland | Cohort study | 200 | 84/114 | 0-20 years | Exclusive BF |
| 63 | Milner et al. | 2004 | USA | Cohort study | 8285 | 4225/4060 | 0-3 years | BF |
| 64 | Wetzig et al. | 2000 | Germany | Cohort study | 475 | NM | 0-1 year | BF |
| 65 | Saarinen et al. | 2000 | Finland | Cohort study | 6209 | NM | 0-1 year | Partial BF |
| 66 | Saarinen et al. | 2000 | Finland | Cohort study | 6209 | NM | 0-1 year | Partial BF |
| 67 | Kull et al. | 2002 | Sweden | Cohort study | 4089 | 2065/2024 | 0-2 years | Exclusive BF |
| 68 | Liao et al. | 2014 | Taiwan,China | Cohort study | 258 | 125/113 | 0-36 months | Exclusive BF |

**Table S2:** Characteristics of the included studies. (Continued)

| Group of BF duration, month | Diagnostic criteria for allergic rhinitis or food allergy | Age of outcome, | Adjusted variables |
| --- | --- | --- | --- |
|  |  |  |  |
| ＞6 months | SFAR | AM:6-12 years | Adjusted |
| ≥6 months | ISAAC | AM:2 and 6 years | Adjusted for sex, maternal age, mean income, first degree relative with allergy, maternal smoking during the child’s first two years and birthweight |
| ＞6 months | SFAR | AM:6-12 years | Adjusted |
| NM | online questionnaire, Physician diagnosis | AM:6-18 years | Adjusted |
| <6, 6-11, ≥12 months | SPT | AM:8.1±2.6 years | Adjusted for the child’s age and sex, number of siblings, mode of delivery, number of siblings, parental atopy history, and living area. |
| 6 months | ISAAC phase I | AM:6-7 years | Adjusted |
| ≥1 month | Questionnaires and physician diagnosis | AM:3 and 6 years | Adjusted |
| (1)＜1, 1-2, 3-6,＞6 months (2) <3, 3-6,＞6 months | ISAAC | AM:4-6 years | Adjusted for family history of atopy, sex, age, district of the current residence, ownership of the current residence, early pet-keeping, early and current parental smoking, as well as early and current home dampness exposure. |
| (1) ≤6, 6 months (2) Never,<3, 3-6, ＞6 months | ISAAC | AM:4-6 years | Adjusted factors included respondent of questionnaire, age, sex, and family history of atopy. |
| ≥6 months | Questionnaires and physician diagnosis | AM:1, 2, 3, 4 years | Adjusted for maternal age at delivery, maternal history ofatopy, paternal history ofatopy, passive smoke exposure from parents during pregnancy, any older siblings, household income, gestational age, and gender |
| ＞6, ≤6 months | ISAAC | AM:3, 4, 5, 6 years | Unadjusted |
| 4 months | ISAAC | AM:9.19±1.76 years | Adjustedfor age, gender, household income per capita, family numbers, resident area per capita, parental education level, city. |
| ＜6,≥6 months | ISAAC | AM:6-7 years | Adjusted |
| NM | ISAAC | AM:10.2±3.3 years | Unadjusted |
| ＜6,≥6 months | CAPI | AM:0-17 years | Each association was adjusted by all the other associations in the model. |
| ＜2, ≥2 months | ISAAC | AM:15-18 years | Adjusted |
| NM | Questionnaires | AM:10.6±2.5 years | Adjusted for age, gender, parental allergic disease and parental education. |
| ≥6 months | ISAAC, SPT | AM:4.37+0.76 years | Adjusted |
| ≥2 months | Questionnaires and physician diagnosis | AM:2, 5 years | Adjusted |
| NM | ISAAC | AM:12.56 years | Adjusted |
| NM | ISAAC Phase Three | AM:6-7 years | Adjusted for sex, region of the world,language, gross national income, birth weight, maternal education, maternal smoking during the child’s first year of life, presence of a cat or dog in the home during the first year of life, paracetamol use during first year of life, antibiotic use during the first year of life, older siblings, maternal exposure to farm animals during pregnancy and exposure to farm animals during the first year of life. |
| <3,3-6,≥6 months | ISAAC | AM:6.5 years | Adjusted |
| (1) ＜4, 4-6, 7-12, 13+months (2) ＜1, 1-3, 4-11, ≥12months | ISAAC phase I | AM:6-8, 9-11, 12-15 years | Adjustment for age, sex, number of siblings, smoking in the household, paternal and maternal history of asthma, atopic eczema, and allergic rhinitis, and paternal and maternal educational level. |
| NM | Questionnaires | AM:6-7, 8-9, 10-12, 13-15 years | adjustment for age,sex, and rural/urban residence |
| ＜6, 6–12, ＞12 months | ISAAC phase I | AM:6-8, 9-14 years | adjusted |
| ＜1, ≥1 month | parental interviews | AM:16 years | adjusted for cohort, sex, and all other factors in table |
| 1 month | Questionnaires | AM:1 month | Adjusted for other factors in the table, sex, and year of birth |
| 4weeks | ISAAC | AM&FA:55 (16-78) d-4 years | adjust for infant’s sex, race and ethnicity, mode of delivery, daycare attendance at enrollment, maternal asthma, exposure to tobacco smoke in utero or prior to enrollment,type of insurance at enrollment,and the presence of another child age ≤6 years living in the same home at enrollment |
| ＜6, ≥6 months | ISAAC, SPT | NM | adjust for gender, ETS exposure, fungi on house walls, and residence location |
| ≤6,＞6 months | ISAAC | AM&FA:6-11 years | adjustment for residential area and whether the address changed after birth |
| NM | ISAAC, SPT | AM&FA:13-14 years | Unadjusted |
| 4 months | ISAAC, SPT | AM&FA:6-7 years | Unadjusted |
| 4.1 (3.1-5.5) months | interviews | FA:7 years | adjusted for reverse causation by excluding children who had eczema,recurrent wheeze, or a positive SPT response during the period of exclusive breast-feeding. |
| NM | ISAAC | FA:7-15 years | Adjusted for sex, age, birth order, family history, eczema or wheeze within 6 months after birth, and FA in infancy. |
| <6 months, ≥6 months | Questionnaires | AM&FA:0-5 years | unadjusted |
| ≥6 months | allergen-specific serum IgE levels | FA:0-4 years | adjusted for child’s sex, gestational age, maternal age at delivery, parental history of atopy, passive smoke exposure, any older siblings, and household income. |
| ＞6 months | Food specific IgE levels | FA:2 months-5 years | sex, age |
| NM | Questionnaires and medical records | FA:6 years | sex, child’s race, child’s ethnicity, maternal smoking status, maternal education, and daycare attendance during infancy |
| NM | Questionnaires | FA:0-21 years | Adjusted for age, sex, maternal race, presence of household pet, and annual household income |
| 3 months | Questionnaires | FA:8 years | child's sex, mode of delivery, maternal age at birth, breastfeeding at maternity, maternal geographic origin, day care at 3 mo, parental socioeconomic status at birth, older siblings at birth, maternal smoking during pregnancy, exposure to environmental tobacco smoke during the first year of life, parental history of asthma, eczema, allergic rhinitis, and food allergy, adverse food reaction in the first 3 mo, eczema symptoms in the first 3 mo, wheezing in the first 3 mo, lower respiratory infection (bronchitis or bronchiolitis) during the first year, body mass index for age and sex at 8 y based on the WHO child growth |
| NM | Skin prick test, Serum-specific IgE to CMPA, Diet elimination, Iron deficiency anemia, Biochemical markers | FA:4.2 ± 3.2 months | Parent's food allergy, Cesarean section, Exclusive breastfeeding, Mixed feeding, Weaning food > 6 months, Weaning food < 4 months, Constant |
| Exclusive breastfeeding: 6-7 months | Questionnaires | FA:18, 66 months | Adjusted for sex, parity, singleton or multiple birth, term or preterm birth, normal or low birth weight, maternal age at delivery, maternal educational attainment, maternal smoking status, place of birth and residence, and daycare attendance. |
| ≤4, 4-6, >6 months | extensive questionnaire and blood sampling | FA:7-10 years | This model was adjusted for comorbid atopy (asthma, allergic rhinitis, or atopic dermatitis in subject or first-degree family member).This model was adjusted for comorbid atopy and IS (specific IgE against birch, grass, mugwort, parietaria, HDM, or cat) |
| NM | Oral food challenges, Skin prick tests, serum IgE | FA:6.25-9.35 years | the time of enrollment, Ara h2 level, breast‐feeding status, and peanut IgE level. |
| ＞6, ≤ 6 months | Questionnaires | NM | birth season, maternal allergic history, maternal age, maternal exposure to smoking, delivery method, presence of siblings, infants’ sex, and infants’ development of atopic dermatitis at 6 months |
| 2, 4 months | Questionnaires, IgE or skin test | FA:2 years | adjusted for parental allergy history, sibling allergy history, age at first attendance at collective care arrangement, first physician seen after discharge, maternal education level and age at first child, parental place of birth, family monthly income per consumption unit, size of town of residence, maternal smoking status, passive smoking, gestational age at birth, sex, mode of delivery, any breastfeeding duration, number of people per room living in the household, recruitment wave, maternity unit size and level, and mother’s region of residence. Among infants with familial history of allergy, analyses were also adjusted for the origin (parents or siblings) of this familial history of allergy. |
| <6, >6-month | Questionnaires | FA:0-18 years | gender, age, region, education level |
| 6.28±5.21 months | DBPCFC | NM | adjusted for the confounding effect of the parental atopic score and a history of asthma |
| NM | IgE, oral food challenge | FA:7.4±7.2 years | Adjusted |
| ≥6, <6 month | Skin prick test, Questionnaires | FA:2-5 years | Adjusted |
| ≥6, <6 month | SPT or specific IgE > 0.35 kU/L, Questionnaires | FA:0–13 years | age and maternal BMI |
| (1) Healthy:4(2-4) (2) Allergic:1(0-3) | Clinical examination, blood tests, open food challenge test and/or a positive Fx5 Food Mix test | FA:1.5, 3 years | maternal heredity, paternal smoking during the last month of pregnancy, cats or dogs in the house at recruitment, gestational week, cesarean section, birth weight, male gender and farm/non-farm family |
| ＜1,1-4,4-6,＞6 months | Clinical data, recruitment questionnaire, IgE, positive provocation test | FA:5.07 ± 2.67 months | pregnancy tolerance, duration of pregnancy, type of delivery, formula feeding in hospital, duration of breastfeeding, and family history of allergy |
| <2, 2-4, 4-6, and ≥6 months | Questionnaires, skin prick tests | FA:10 years | Models are adjusted for maternal education, history of allergy, eczema, or asthma, parity, pet keeping, body mass index (BMI) at enrollment, smoking, psychiatric symptoms, and child’s sex, gesta-tional age, birthweight, ethnic origin, and day care attendance, and mutually for inhalant and food-allergic sensitization or allergy. |
| 0, 1 , 2 ,3, 4, 5, 6, ＞6 months | Skin prick test, open food challenge test | FA:1 year | family history of allergy, infant eczema status during breastfeeding, parent reported reactions to cow’s milk in the infant and age of introduction of egg |
| 6 months | symptoms, specific IgE | FA:(1) Children with polysensitization: 1.6±1.5years (2) Children with monosensitization: 1.8±1.8years | sex (male), age (year), paren­tal history of allergic rhinitis, comorbid allergic diseases, season of birth (summer/fall), and diet in the first 6 months of age (exclusive breastfeeding) |
| 0, 1-3, ≥4 months | Questionnaires | FA:1, 6 years | higher maternal education and family income, family history of food allergy, and reported eczema before age 1 year |
| <0.5, 0.5-3, ＞3 months | Serum IgE antibodies | FA:5 years | sex of the child, number of siblings, parental asthma, parental rhinitis, hospital of birth, maternal smoking during pregnancy) and based on statistical tests (season of birth, duration of gestation, maternal age, maternal basic education, pets at home by 1 year, mode of delivery, and birth weight |
| ≥4 months | The cord blood total IgE | FA:6 to 12 months and 2, 4 and 6 years | maternal age at delivery, family history of allergy, maternal education, maternal smoking during pregnancy, maternal smoking after delivery, child’s age when FS was defined, sex, ancestral proportion, pets in the first year, and allergic disease during the first 4 months of life |
| 90 (105) days | a provocation test withAF, specific IgE antibodies to casein, alphalactoalbumin and beta-lactoglobulin | FA:4 (3.5) months | Adjusted |
| NM | Questionnaires, a skin prick test or a blood radioallergosorbent immunoassay test | FA:5 years | age and sex |
| <2, 2-6,6-9, ≥9 months | Skin prick testing,clinical examination,and parental and personal structured interviews | FA:1, 5, 11, 20 years | duration of exclusive breastfeeding (＜9 months/≥9 months), sex, maternal age, maternal educational level, smoking in the household during the first year, day care attendance at the age of 1 year, sibship size 3 or more. |
| NM | parental report of physician diagnosis of asthma or food allergies | FA:3 years | Adjusted |
| ＞5 months | cord blood IgE, Questionnaires | FA:1 year | length of breast-feeding, sensitization and other risk factors |
| 7.5 months or longer | open cow's milk challenge, skin-prick tests, Total IgE and specific IgE cow's milk antibodies | FA:6.7 months | age at first exposure to cow's milk at home, a family history of atopy, maternal smoking and the presence of siblings and furred household pets |
| (1)IgE-mediated: 8.4(7.8,9.1) (2)Non-IgE-mediated4.5(3.5,5.7) | open cow's milk challenge, skin-prick tests, Total IgE and specific IgE cow's milk antibodies | FA:6.7 (2.8-12.7) months | Adjusted |
| <4, ≥4 months | Clinical examination, symptoms | FA:1, 2 years | gender, heredity for allergic disease, mother's age, maternal smoking during pregnancy and/or during the first months of life of the baby, and year of construction of the home |
| <4, ≥4 months | absolute eosinophil count, total IgE level, and specific IgE | FA:6, 12, 18, 24 and 36 months | gender, mode of delivery, birth body weight, smoking during pregnancy, maternal education, house pet exposures, elevated cord IgE, early eczema, and paternal or maternal history of allergy |

**Notes:** Data are shown as numbers, mean ± SD, median (IQR) or mean (range). AR, Allergic rhinitis; FA, Food allergy; SFAR, Score for Allergic Rhinitis; ISAAC, The International Study of Asthma and Allergies in Childhood; SPT, skin prick test; CAPI, Computer-assisted personal interview; DBPCFC, double-blind placebo-controlled food challenge; NM, Not mentioned.

**Table S3:** Quality assessment of included studies (Newcastle Ottawa Scale).

| **Author** | **Year** | **Selection** | | | | **Comparability** | **Outcome** | | | **Quality scores** |
| --- | --- | --- | --- | --- | --- | --- | --- | --- | --- | --- |
|  |  | Representativeness of the exposed cohort | Selection of the nonexposed cohort | Ascertainment of exposure | Demonstration that outcome of interest was not present at start of study | Comparability of cohorts on the basis of the design or analysis^&^ | Assessment of outcome | Was follow-up long enough for outcomes to occur^#^ | Adequacy of follow up of cohorts^¶^ |  |
| Rosas-Salazar et al. | 2022 | **★** | **★** | **★** | **★** | **★★** | **★** | **★** | **-** | 8 |
| Sung et al. | 2022 | **★** | **★** | **★** | **★** | **★★** | **★** | **★** | **-** | 8 |
| Ekelund et al. | 2021 | **★** | **★** | **★** | **★** | **★★** | **★** | **★** | **★** | 9 |
| Pelak et al. | 2021 | **★** | **★** | **★** | **★** | **★★** | **★** | **★** | **★** | 9 |
| Lang et al. | 2021 | **★** | **★** | **★** | **★** | **★★** | **★** | **★** | **★** | 9 |
| Amazouz et al. | 2021 | **★** | **★** | **★** | **★** | **★★** | **★** | **★** | **★** | 9 |
| Matsumoto et al. | 2020 | **★** | **★** | **★** | **★** | **★★** | **★** | **★** | **★** | 9 |
| Sicherer et al. | 2019 | **★** | **★** | **★** | **★** | **★★** | **★** | **★** | **★** | 9 |
| Kim et al. | 2019 | **★** | **★** | **★** | **★** | **★★** | **-** | **★** | **★** | 8 |
| Davisse-Paturet et al. | 2019 | **★** | **★** | **★** | **★** | **★★** | **★** | **★** | **-** | 8 |
| van Ginkel et al. | 2018 | **★** | **★** | **★** | **★** | **★★** | **★** | **★** | **★** | 9 |
| Sardecka et al. | 2018 | **★** | **★** | **★** | **★** | **★★** | **-** | **★** | **★** | 8 |
| Bedolla-Barajas et al. | 2018 | **★** | **★** | **-** | **★** | **★★** | **★** | **★** | **★** | 8 |
| Alkazemi et al. | 2018 | **★** | **★** | **★** | **★** | **★★** | **★** | **★** | **★** | 9 |
| Lee et al. | 2017 | **★** | **★** | **★** | **★** | **★★** | **★** | **★** | **-** | 8 |
| Jonsson et al. | 2017 | **★** | **★** | **★** | **★** | **★★** | **★** | **★** | **★** | 9 |
| Gil et al. | 2017 | **★** | **★** | **★** | **★** | **★★** | **★** | **★** | **★** | 9 |
| Elbert et al. | 2017 | **-** | **★** | **★** | **★** | **★★** | **★** | **★** | **★** | 8 |
| Chiu et al. | 2016 | **-** | **★** | **★** | **★** | **★★** | **★** | **★** | **-** | 7 |
| Goldsmith et al. | 2016 | **-** | **★** | **★** | **★** | **★★** | **★** | **-** | **★** | 7 |
| Jelding-Dannemand et al. | 2015 | **★** | **★** | **★** | **★** | **★★** | **★** | **★** | **★** | 9 |
| Kim et al. | 2015 | **★** | **★** | **★** | **★** | **★★** | **★** | **★** | **★** | 9 |
| Luccioli et al. | 2014 | **★** | **★** | **★** | **★** | **★★** | **★** | **★** | **★** | 9 |
| Liao et al. | 2014 | **★** | **★** | **★** | **★** | **★★** | **★** | **★** | **★** | 9 |
| Nwaru et al. | 2013 | **-** | **★** | **★** | **★** | **★★** | **★** | **★** | **★** | 8 |
| Kellberger et al. | 2012 | **★** | **★** | **-** | **★** | **★★** | **★** | **★** | **-** | 7 |
| Sandini | 2011 | **★** | **★** | **★** | **★** | **★★** | **★** | **★** | **★** | 9 |
| Hong et al. | 2011 | **★** | **★** | **★** | **★** | **★★** | **★** | **★** | **★** | 9 |
| Kramer et al. | 2009 | **★** | **★** | **★** | **★** | **★★** | **★** | **★** | **★** | 9 |
| Sanchez-Valverde et al. | 2009 | **★** | **★** | **★** | **★** | **★★** | **★** | **★** | **★** | 9 |
| Pesonen et al. | 2006 | **★** | **★** | **★** | **★** | **★★** | **★** | **★** | **★** | 9 |
| Milner et al. | 2004 | **★** | **★** | **★** | **★** | **★★** | **-** | **★** | **★** | 8 |
| Kull et al. | 2002 | **★** | **★** | **★** | **★** | **★★** | **★** | **★** | **★** | 9 |
| Wetzig et al. | 2000 | **★** | **★** | **★** | **★** | **★★** | **★** | **-** | **★** | 8 |
| Saarinen et al. | 2000 | **★** | **★** | **★** | **★** | **★★** | **★** | **-** | **★** | 8 |
| Saarinen et al. | 2000 | **★** | **★** | **★** | **★** | **★★** | **★** | **-** | **★** | 8 |
| Butland et al. | 1997 | **★** | **★** | **★** | **★** | **★★** | **★** | **★** | **-** | 8 |
| Strachan et al. | 1996 | **★** | **★** | **★** | **★** | **★★** | **★** | **★** | **-** | 8 |

**Table S4:** Joanna Briggs Institute Critical Appraisal tool for cross-sectional study.

| **Study** | **Q1** | **Q2** | **Q3** | **Q4** | **Q5** | **Q6** | **Q7** | **Q8** | **Quality** |
| --- | --- | --- | --- | --- | --- | --- | --- | --- | --- |
| Tong et al. | Yes | Yes | Yes | Yes | Yes | Unclear | Yes | Yes | High |
| Tong et al. | Yes | Yes | Yes | Yes | Yes | Unclear | Yes | Yes | High |
| Yu et al. | Unclear | Yes | Yes | No | Yes | Unclear | Yes | Yes | Moderate |
| Han et al. | Yes | Yes | Yes | Yes | Yes | Yes | Yes | Yes | High |
| Chinratanapisit et al. | Yes | Yes | Yes | No | Yes | Unclear | Yes | Yes | Moderate |
| Huang et al. | Yes | Yes | Yes | No | Yes | Yes | Yes | Yes | High |
| Wang et al. | Yes | Yes | Yes | No | Yes | Yes | Yes | Yes | High |
| Wang et al. | Yes | Yes | Yes | No | No | No | Yes | Yes | Moderate |
| Li et al. | Yes | Yes | Yes | No | Yes | Yes | Yes | Yes | High |
| Tamay et al. | Yes | Yes | Yes | No | Yes | Unclear | Yes | Yes | Moderate |
| Song et al. | Yes | Yes | Yes | No | No | No | Yes | Yes | Moderate |
| Schmitz et al. | Yes | Yes | Yes | No | Yes | Yes | Yes | Yes | High |
| Chen et al. | Yes | Yes | Yes | No | Yes | Yes | Yes | Yes | High |
| Siriaksorn et al. | Yes | Yes | Yes | Yes | Yes | Unclear | Yes | Yes | High |
| Sahebi et al. | Unclear | Yes | Yes | No | Yes | Unclear | Yes | Yes | Moderate |
| Bjorksten et al. | Yes | Yes | Yes | No | Yes | Yes | Yes | Yes | High |
| Miyake et al. | Yes | Yes | Yes | No | Yes | Yes | Yes | Yes | High |
| Kurt et al. | Unclear | Yes | Yes | No | Yes | Yes | Yes | Yes | Moderate |
| Obihara et al. | Yes | Yes | Yes | No | Yes | Unclear | Yes | Yes | Moderate |
| Huang et al. | Unclear | Yes | Yes | Yes | Yes | Unclear | Yes | Yes | Moderate |
| Hu et al. | Yes | Yes | Yes | No | Yes | Yes | Yes | Yes | High |
| Ahmed et al. | No | Yes | Yes | Yes | No | No | Yes | Yes | Moderate |
| Ahmed et al. | No | Yes | Yes | Yes | No | No | Yes | Yes | Moderate |
| Kusunoki et al. | Yes | Yes | Yes | No | Yes | Yes | Yes | Yes | High |
| Ehlayel et al. | Yes | Yes | Yes | No | No | No | Yes | Yes | Moderate |
| Yuenyongviwat et al. | Yes | Yes | Unclear | Yes | Yes | Yes | Yes | Yes | High |
| Saad et al. | Yes | Yes | Unclear | Yes | Yes | Yes | Yes | Yes | High |
| Lyons et al. | Yes | Yes | Unclear | Yes | Yes | Yes | Yes | Yes | High |
| Wang et al. | No | Yes | Unclear | Unclear | Yes | Yes | Yes | Yes | Moderate |
| Kljakovic et al. | Unclear | Yes | Unclear | Yes | Yes | Yes | Yes | Yes | Moderate |

The JBI Checklist provides quality criteria for prevalence studies in eight distinct items. The table indicates which items have been fulfilled for each included study, respectively:

Q1: Were the criteria for inclusion in the sample clearly defined?

Q2: Were the study subjects and the setting described in detail?

Q3: Was the exposure measured in a valid and reliable way?

Q4: Were objective, standard criteria used for measurement of the condition?

Q5: Were confounding factors identified?

Q6: Were strategies to deal with confounding factors stated?

Q7: Were the outcomes measured in a valid and reliable way?

Q8: Was appropriate statistical analysis used?

**
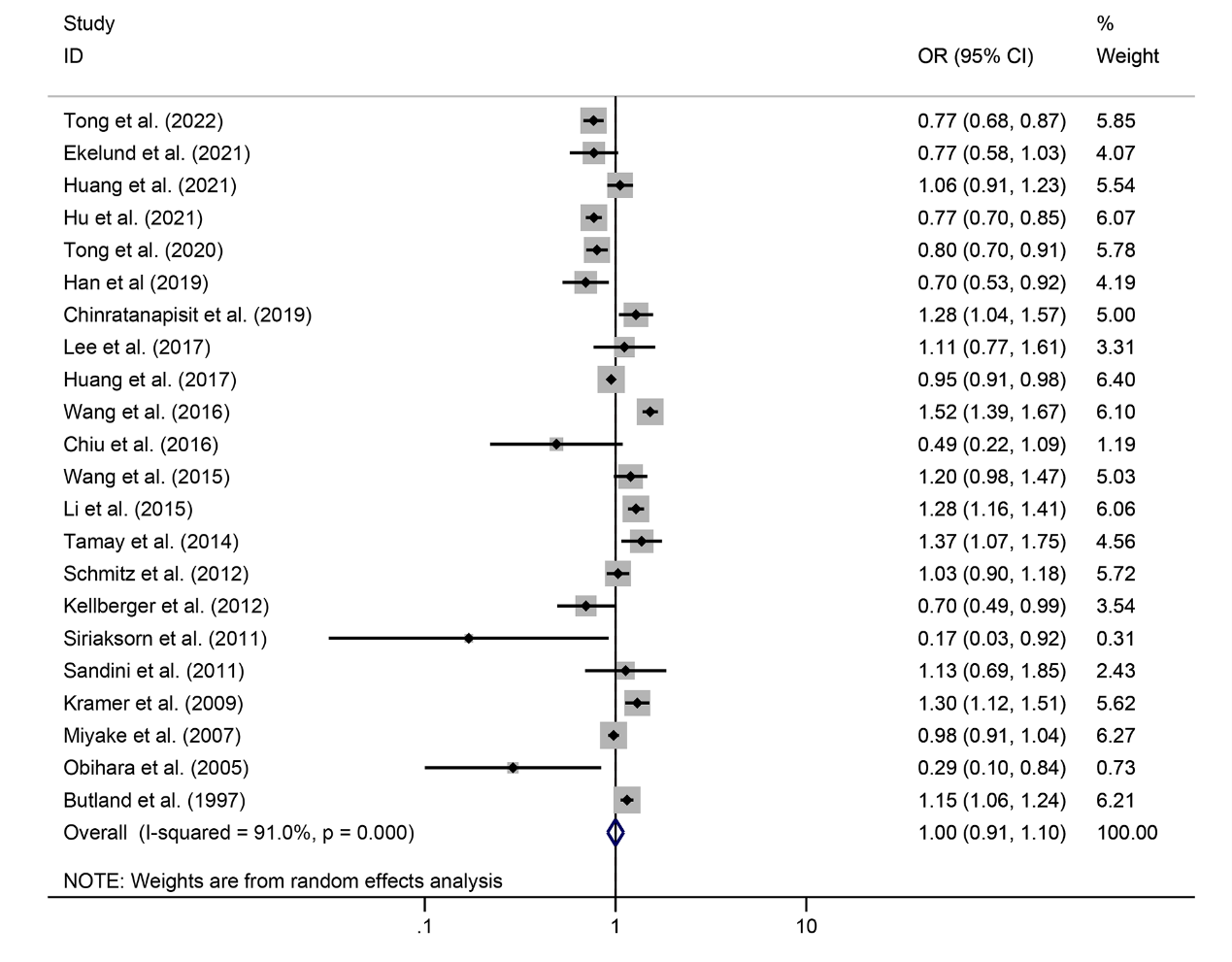
**

**Figure S1:** Forest plot of the relationship between the duration of breastfeeding and allergic rhinitis in offspring.


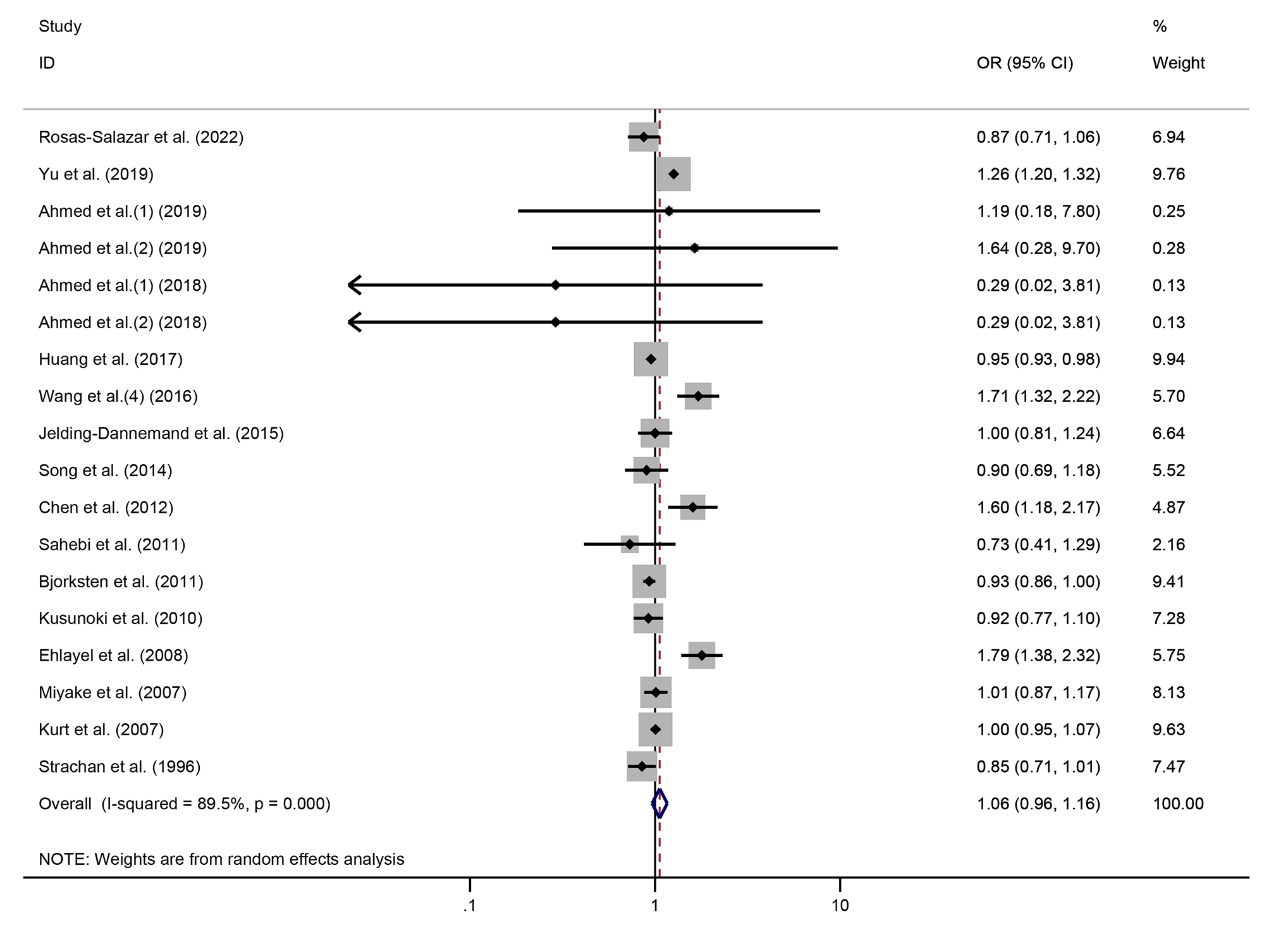


**Figure S2:** Forest plot of the relationship between the pattern of breastfeeding and allergic rhinitis in offspring.


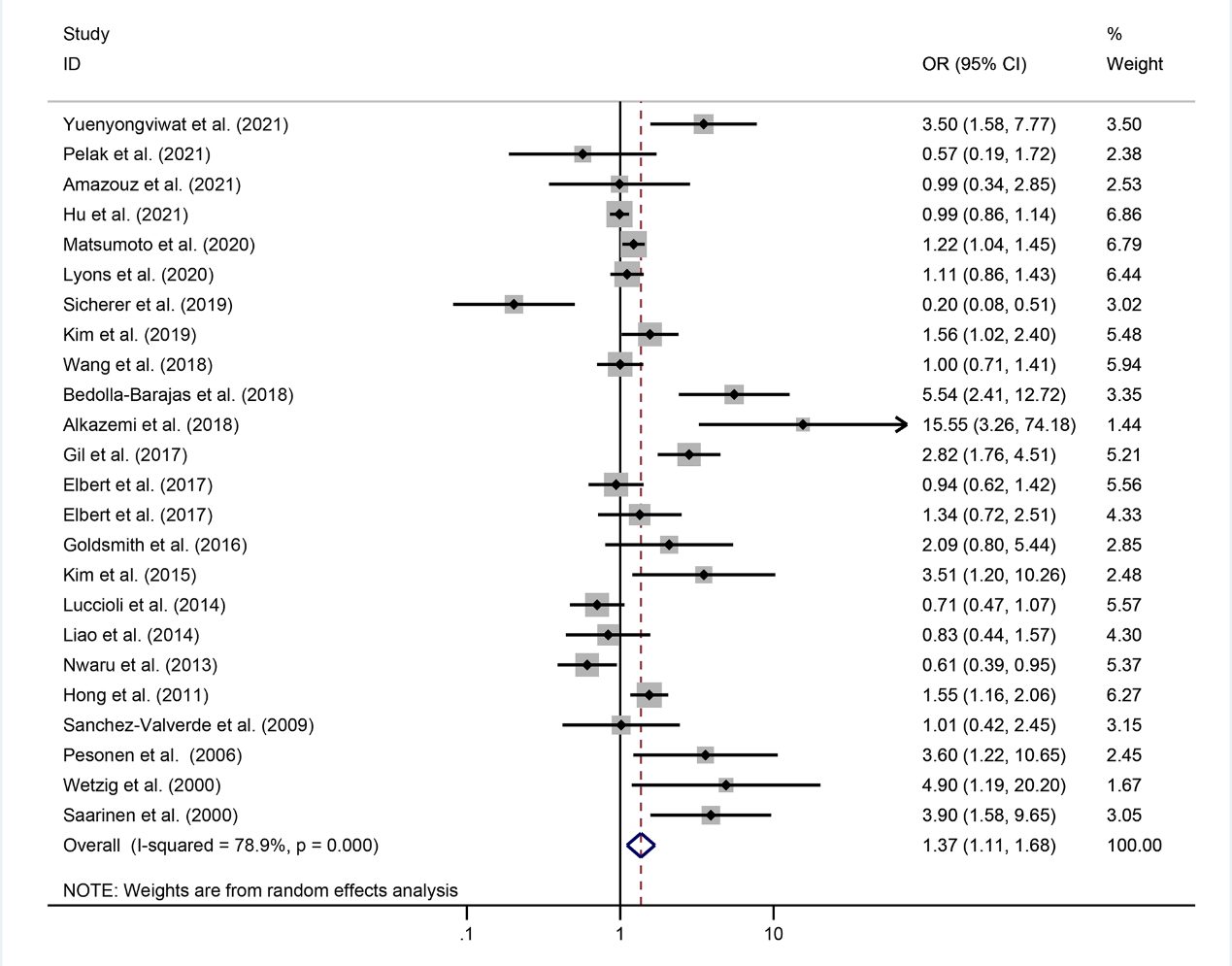


**Figure S3:** Forest plot of the relationship between the duration of breastfeeding and food allergy in offspring.


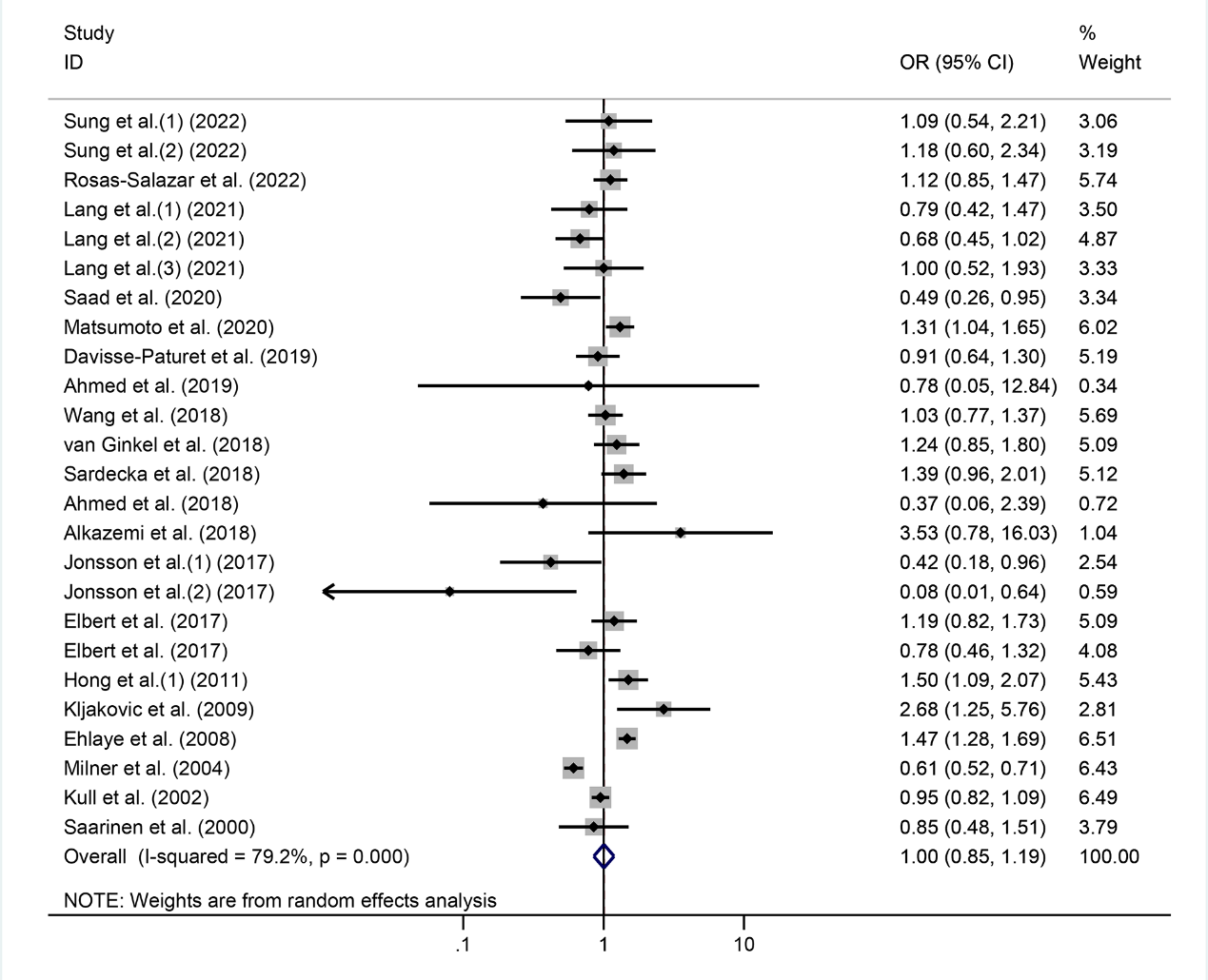


**Figure S4:** Forest plot of the relationship between the pattern of breastfeeding and food allergy in offspring.


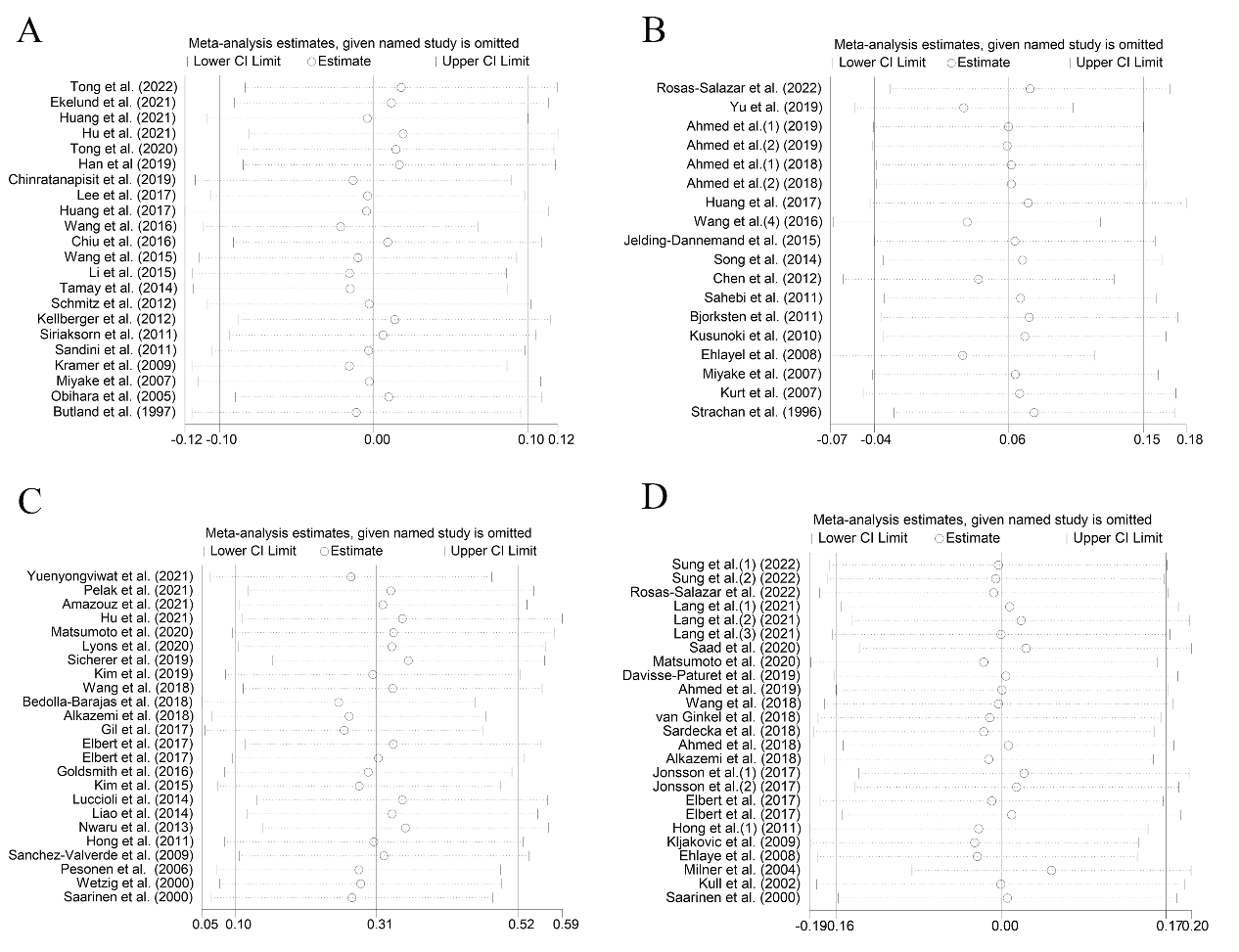


**Figure S5:** Sensitivity analysis for the influences of the duration and pattern of breastfeeding on allergic rhinitis and food allergy by sequentially excluding each individual study. (A) The influences of the duration of breastfeeding on allergic rhinitis; (B) The influences of the pattern of breastfeeding on allergic rhinitis; (C) The influences of the duration of breastfeeding on food allergy; (D) The influences of the pattern of breastfeeding on food allergy.
